# Enhancing X-ray Attenuation of 3D Printed Gelatin Methacrylate (GelMA) Hydrogels Utilizing Gold Nanoparticles for Bone Tissue Engineering Applications

**DOI:** 10.3390/polym11020367

**Published:** 2019-02-20

**Authors:** Nehar Celikkin, Simone Mastrogiacomo, X. Frank Walboomers, Wojciech Swieszkowski

**Affiliations:** 1 Faculty of Material Science and Engineering, Warsaw University of Technology, 00-661 Warszawa, Poland; neharcelikkin@gmail.com; 2 Radboud University Medical Center, Department of Biomaterials, Philips van Leijdenlaan 25, 6525 EX, Nijmegen, The Netherlands; simone.mastrogiacomo@nih.gov (S.M.); frank.walboomers@radboudumc.nl (X.F.W.); 3 Laboratory of Functional and Molecular Imaging, NINDS, National Institutes of Health, Building 10, 5S261, Bethesda, MD 20892, USA

**Keywords:** 3D printed gelatin methacrylate hydrogels, gold nanoparticles, µCT imaging, contrast agent for CT imaging

## Abstract

Bone tissue engineering is a rapidly growing field which is currently progressing toward clinical applications. Effective imaging methods for longitudinal studies are critical to evaluating the new bone formation and the fate of the scaffolds. Computed tomography (CT) is a prevailing technique employed to investigate hard tissue scaffolds; however, the CT signal becomes weak in mainly-water containing materials, which hinders the use of CT for hydrogels-based materials. Nevertheless, hydrogels such as gelatin methacrylate (GelMA) are widely used for tissue regeneration due to their optimal biological properties and their ability to induce extracellular matrix formation. To date, gold nanoparticles (AuNPs) have been suggested as promising contrast agents, due to their high X-ray attenuation, biocompatibility, and low toxicity. In this study, the effects of different sizes and concentrations of AuNPs on the mechanical properties and the cytocompatibility of the bulk GelMA-AuNPs scaffolds were evaluated. Furthermore, the enhancement of CT contrast with the cytocompatible size and concentration of AuNPs were investigated. 3D printed GelMA and GelMA-AuNPs scaffolds were obtained and assessed for the osteogenic differentiation of mesenchymal stem cells (MSC). Lastly, 3D printed GelMA and GelMA-AuNPs scaffolds were scanned in a bone defect utilizing µCT as the proof of concept that the GelMA-AuNPs are good candidates for bone tissue engineering with enhanced visibility for µCT imaging.

## 1. Introduction

Bone tissue engineering (BTE) is a rapidly growing field, with the research being conducted in various fields, including engineering, pharmaceutics, and medicine [1,2]. The scope of bone tissue engineering has recently expanded beyond in vitro and animal studies, and is currently progressing toward clinical applications. Thus, effectual imaging methods for longitudinal studies are critical to evaluating the outcomes of tissue engineered constructs. For instance, even though histological techniques provide crucial information, these destructive imaging techniques are incapable of providing 3D information, which significantly hinders the use of such conventional methods for in vivo and preclinical applications [3]. Therefore, an increasing number of recent tissue engineering studies feature the compatibility of the utilized 3D scaffolds with different advanced imaging modalities [4,5,6,7].

Radiography is the second most widely used imaging modality thanks to its good spatial resolution (50–200 μm), short acquisition time (<10–15 min), and availability. Computed tomography (CT) is used in the staging and imaging-guided intervention of various diseases, producing 3D information regarding its non-invasive nature, a high resolution, and deep tissue penetration [8]. Besides its use in diagnostics, CT is an ascendant technique used to evaluate the performance of tissue implants and tissue engineering scaffolds [9,10,11]. Furthermore, the use of micro-CT (μCT) enables researchers to attain voxels with a micrometer spatial resolution, thus it is able to obtain high-resolution volumetric images in relatively short scan times specifically for hard tissue regeneration and scaffold degradation [9,10,11,12,13].

In CT imaging, the X-ray attenuation of a material is proportionally related to its atomic number and density; thereby, the CT contrast is high in mineralized tissues (e.g., bone and teeth), while it becomes low in mainly-water containing tissues (i.e., soft tissues). For the same reasons, hydrogels, one of the most prominent materials used in tissue engineering applications, show little or no contrast on CT images as they mainly consist of water. Enhancement of the CT contrast can be achieved by using specific contrast agents (CA). To date, gold nanoparticles (AuNPs) have been suggested as promising CA, as they show high X-ray attenuation (5.16 cm^2^/g at 100 keV), biocompatibility, low toxicity, high chemical stability, and narrow size control during synthesis. Moreover, AuNPs have shown the ability to enhance CT contrast of ceramic-based bone substitutes, as well as to the capability induce osteoblast differentiation and new bone formation [6,14,15].

Among different hydrogels used for bone tissue engineering applications, gelatin methacrylate (GelMA) hydrogels are widely used for tissue regeneration due to their optimal biological properties and their ability to induce extracellular matrix formation [16,17,18]. Recent insights indicate that GelMA can specifically induce in vitro osteogenic differentiation and calcium deposition, as well as endochondral bone formation in vivo*,* hence supporting its potential use in BTE [19,20,21]. Besides desirable biological characteristics, the methacrylate moieties on GelMA’s backbone give rise to a stable crosslinked hydrogel with rapid permanent crosslinking when exposed to UV light and in the presence of a suitable photoinitiator. Thus, GelMA hydrogels are suitable materials for different manufacturing techniques, such as photopatterning, micromolding, and layer-by-layer 3D printing [22].

Layer-by-layer 3D printing is one of the most convenient techniques used to create TE-dedicated scaffolds with well-distributed and interconnected pores which ensure cell migration, vascular in-growth, and nutrient diffusion, as well as the removal of waste products [2,23,24,25,26]. Furthermore, printing hydrogels with this technique allows the creation of patient-specific designed scaffolds with structural complexity, low-cost, and high-efficiency [27].

**Figure 1:**
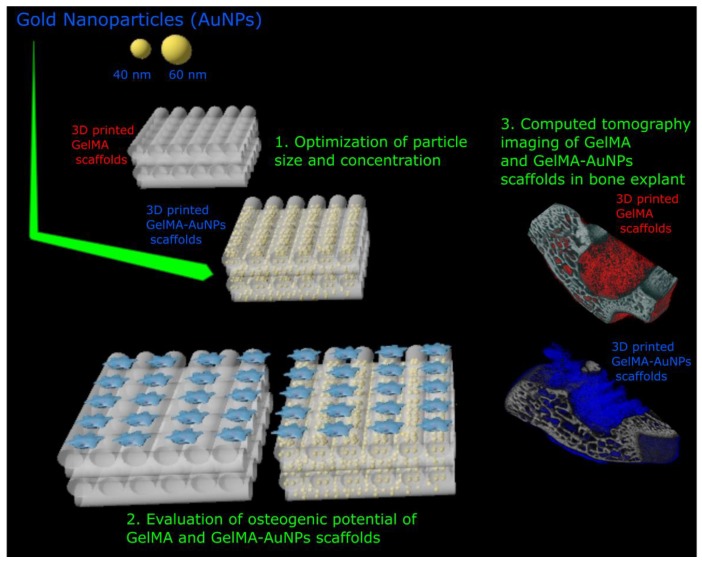
The study design of enhancing X-Ray attenuation of 3D printed gelatin methacrylate (GelMA) hydrogels utilizing gold nanoparticles for bone tissue engineering applications.

In this study, we evaluated the effect of different sizes and concentrations of AuNPs on the mechanical properties and cytocompatibility of the GelMA-AuNPs scaffolds. Furthermore, we have investigated the enhancement of CT signaling with the cytocompatible size and concentration of AuNPs. We have printed 3D GelMA and GelMA-AuNPs scaffolds and assessed the effect of the GelMA-AuNPs on MSC osteogenic differentiation. Lastly, the GelMA and GelMA-AuNPs scaffolds were inserted into a bone defect and µCT indicated as the proof of concept that the GelMA-AuNPs are good candidates for bone tissue engineering with enhanced visibility for µCT imaging (Figure 1).

## 2. Materials and Methods

### 2.1. Preparation of Gelatin Methacrylate (GelMA), GelMA, and GelMA-AuNPs Pre-polymer Solution

GelMA was synthesized as described elsewhere [22]. 10% (w/v) gelatin type A porcine skin (Sigma-Aldrich, St. Louis, MO, USA) was dissolved in phosphate buffer solution (PBS) at 60 °C and after complete dissolution, 800 µL methacrylic anhydride (Sigma-Aldrich, Poznan, Poland) per gram of gelatin was added under constant stirring. After the reaction, the mixture was dialyzed against distilled water using 12–14 kDa cut-off dialysis tubing (Spectra/Por 1 Dialysis Membranes, Spectrum Labs, Rancho Dominguez, CA, USA) to remove the residual salts and methacrylic anhydride, and the solution was lyophilized at −80 °C.

The pre-polymer solution of GelMA was prepared at a 5% concentration of GelMA and 0.05% of the photoinitiator 2-hydroxy-4′-(2-hydroxyethoxy)-2-methylpropiophenone (Sigma-Aldrich, Poznan, Poland) in PBS at 37 °C.

To enhance the CT contrast of GelMA-based scaffolds, AuNPs (Sigma-Aldrich, St. Louis, MO, USA) stabilized by citrate in 0.1 mM PBS, with different sizes and concentrations, were added to the GelMA pre-polymer solution. Specifically, different concentrations of AuNPs were calculated as 0.08 mM, 0.16 mM, and 0.40 mM. These same concentrations were used for two AuNP sizes, i.e., 40 nm and 60 nm.

### 2.2. Evaluation of in vitro Cytocompatibility, Mechanical Properties, and µCT Visibility of GelMA and GelMA-AuNP Bulk Hydrogels

To assess the influence of AuNPs on in vitro cytocompatibility, the mechanical properties, and µCT visibility of GelMA hydrogel, cylindrical discs of GelMA and GelMA-AuNPs (diameter = 10 mm, thickness = 1 mm, *n* = 5) were prepared from pre-polymer solutions. In this study, 25 µL prepolymer solutions of GelMA and GelMA-AuNPs were pipetted into a PDMS mold and were then exposed to UV light at 12.5 mW/cm^2^ UV light (BlueWave^®^ 75 UV Light Curing Spot Lamp, 365 nm, Torrington, CT) for 60 s for permanent crosslinking.

To evaluate the acute cytotoxic effect of AuNP size and concentration, GelMA-AuNP hydrogels (prepared under sterile conditions) were seeded with L929 fibroblasts (mouse C3H/An, ECACC, UK) and cultured in Dulbecco’s Modified Eagle Medium (DMEM, Gibco, Grand Island, MA, USA), 10% FBS (EuroClone S.p.A., Pero, Italy), and 100 µg/mL Penicillin-Streptomycin (10,000 U/mL, Gibco, Bleiswijk, The Netherlands) at 37 °C and 5% CO_2_ for 72 h, according to standard practice UNI EN ISO 10993-5. Subsequently, the CellTiter Cell Proliferation Assay (MTS, Promega, Fitchburg, WI) was performed to colorimetrically measure the metabolic activity (FLUOstar Omega UV/Vis spectrometer, BMG LabTech, Ortenberg, Germany) of the fibroblasts cultured on GelMA (i.e., non-labeled) and the GelMA-AuNPs (*n* = 3).

Prior to the mechanical testing, the crosslinking of the hydrogels was evaluated in terms of gel fraction and swelling from a dry and wet state (Figure S1) [28]. To assess the mechanical properties, GelMA and GelMA-AuNPs hydrogels were detached from the molds and tested at a rate of 20% strain/min on a DMA Q800 apparatus (TA Instruments, Warsaw, Poland). The compressive modulus was determined as the slope of the linear region corresponding to 0–5% strain (*n* = 5).

Radiopacity assessments of the GelMA-AuNPs hydrogels containing different sizes of AuNPs in different concentrations were carried out using a Skyscan 1172 (Bruker, Kontich, Belgium) micro CT (µCT) system with the following settings: 35 kV, 800 µA, pixel size of 10.7 µm. Acquired files were reconstructed through NRecon (Skyscan, Kontich, Belgium) reconstruction software, while assessment of the gray value of the hydrogels was performed using CTAnalyser software (version 1.10.1.0, Skyscan, Kontich, Belgium).

### 2.3. 3D Printing of GelMA and GelMA-AuNP Scaffolds

GelMA pre-polymer solution was prepared as explained in Section 2.1, sterile filtered (PES membrane, 0.22 µm), and transferred into the sterile plotting cartridge (Nordson EFD, Westlake, OH). The plotting cartridge inserted into the printing head of a 3D Bioplotter (Envisiontec, Gladbeck, Germany) and the GelMA and GelMA-AuNPs solutions were cooled down to 20 °C. The pre-polymer solutions were extruded through a 23G needle with an inner diameter of 330 µm and a length of 3.81 cm (general purpose dispersing tips, Nordson EFD, East Providence, RI, USA) onto the printing plate, which was cooled down to 4 °C. Layer-by-layer printing, a speed of 18 mm/s, a 2.3 bar extrusion pressure, and a 200 µm layer thickness were used to form 0°/90° oriented strands with a 1.2 mm distance. CAD files specifying the cylindrical geometry (diameter: 30 mm, thickness: 3 mm) of the scaffolds were used as the input to produce the physical model for the 3D Bioplotter (EnvisionTech GmbH, Gladbeck, Germany) and converted into G-code via Visual Machines software (EnvisionTech GmbH, Gladbeck, Germany) for the printing process. 3D constructs were printed and then exposed to 12.5 mW/cm^2^ UV light (BlueWave^®^ 75 UV Light Curing Spot Lamp, 365 nm, Torrington, CT, USA) for 60 s for permanent crosslinking.

### 2.4. In vitro Evaluation of Osteogenic Differentiation of MSCs on GelMA and GelMA-AuNPs

Rat MSCs isolated from six-week-old male Wistar rats (Charles River, Leiden, The Netherlands) were suspended in growth medium and cultured in an incubator at 37 °C and 5% CO_2_ until they reached 80% confluence. Afterward, adherent cells were detached by using trypsin/EDTA (0.25% (w/v) trypsin, 0.02% EDTA, Gibco, Bleiswijk, The Netherlands) counted and seeded onto the scaffolds. 3D printed GelMA (containing 5% (w/v) GelMA) and GelMA-AuNP (containing 5% (w/v) GelMA and 0.16 mM 60 nm AuNPs) scaffolds were prepared as explained in Section 2.3 and cut into even scaffolds using 6 mm sterile biopsy punch. Isolated MSCs were seeded at a density of 10^5^ cells/cm^2^ on 3D printed scaffolds. Scaffolds were cultured in osteogenic medium (α-MEM (Gibco, Carlsbad, CA, USA), 10% fetal FBS (Gibco, Bleiswijk, The Netherlands), 50 mg/mL ascorbic acid (Sigma Aldrich, Poznan, Poland), 10 mM β-glycerolphosphate (Sigma Aldrich, Poznan, Poland), 10^−8^ M dexamethasone (Sigma Aldrich, Poznan, Poland), and 50 mg/mL gentamycin (Gibco, Bleiswijk, The Netherlands) at 37 °C and 5% CO_2_ over 28 days. At different time points (i.e., day 7, day 14, day 21, and day 28), the scaffolds (*n* = 5) were washed in PBS and cells were lysed through freeze-thaw cycles. Supernatants were collected for DNA–Alkaline Phosphatase (AP) quantification and scaffolds were further incubated overnight in 1 mL of 0.5 M acetic acid solution to dissolve calcium (Ca) ions for Ca quantification. The QuantiFluorVR dsDNA system (Promega Corporation, Madison, WI, USA) was used to measure the total amount of DNA, which was determined fluorometrically at an excitation wavelength of 485 nm and an emission wavelength of 530 nm. The AP activity was measured colorimetrically by using *p*-nitrophenyl phosphate as the substrate. The absorbance was measured at 405 nm using a spectrophotometer. AP activity was directly converted from the absorbance and then correlated according to the DNA content of the scaffolds. The calcium content of the scaffolds was then determined using lysates in acetic acid and the *ortho*-cresolphthalein complexone (OCPC method). The absorbance was measured at 570 nm.

### 2.5. In Vitro Imaging of the GelMA and GelMA-AuNP Scaffolds in µCT

3D printed GelMA and GelMA-AuNPs scaffolds were cut into 3 mm scaffolds using a biopsy punch and inserted into a rat condyle bone defect (3 mm diameter). Radiopacity assessments were carried out using a Skyscan 1174 (Bruker, Kontich, Belgium) micro CT (µCT) system with the following settings: 50 kV, 800 µA, pixel size of 10.7 µm. Acquired files were reconstructed through NRecon (Skyscan, Kontich, Belgium) reconstruction software, while assessment of the gray value of the hydrogels was performed using CTAnalyser software (version 1.10.1.0, Skyscan, Kontich, Belgium).

### 2.6. Statistical Method

Samples were tested in triplicate or quintuplicate, and data are reported as the mean value ± standard deviation. Statistical analysis was performed using ANOVA followed by Sidak’s multiple comparison test using GraphPad Prism 5.00 software (La Jolla, CA, USA). Difference was considered statistically significant if *p* < 0.05 (*), *p* < 0.01 (**), and *p* < 0.001 (***).

## 3. Results

### 3.1. Characterization of GelMA-AuNPs Bulk Hydrogel

**Figure 2:**
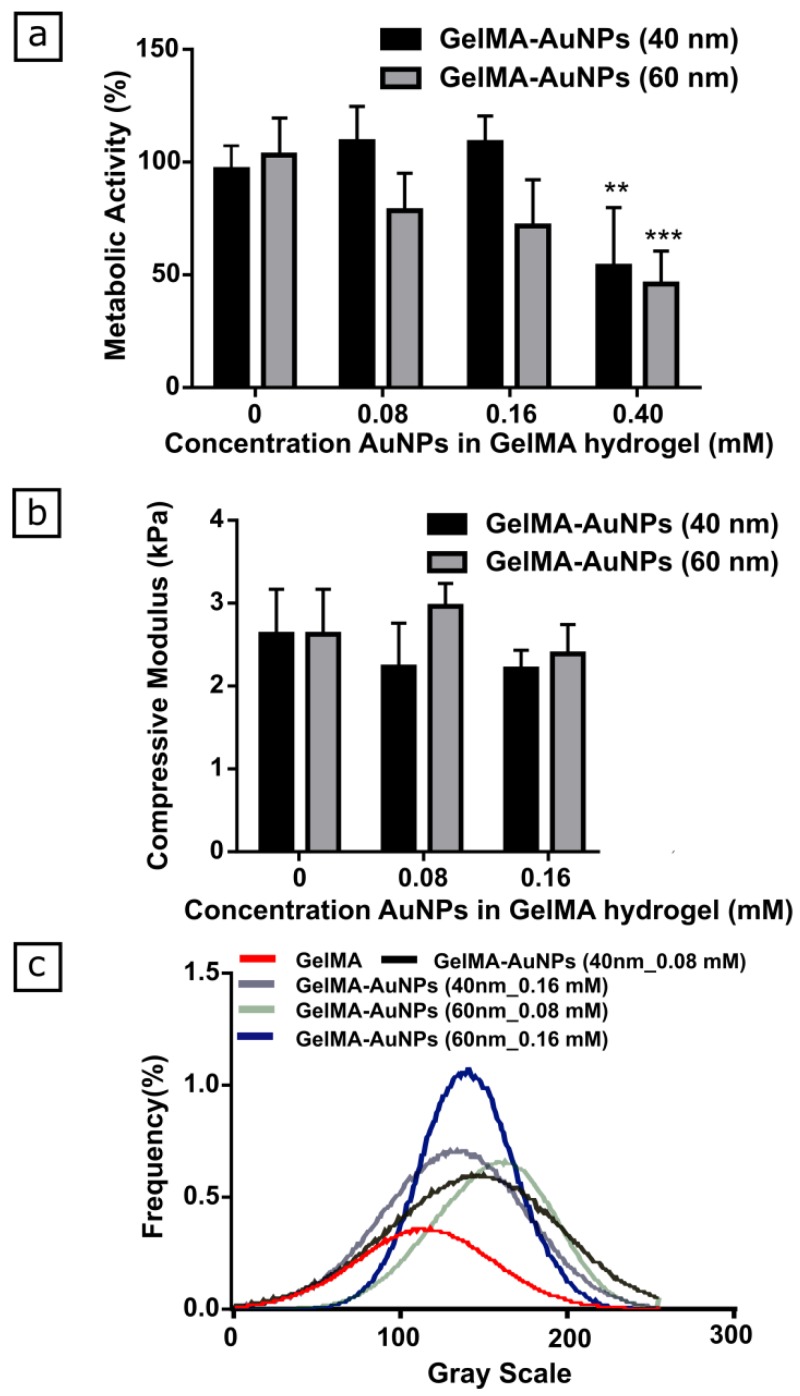
Evaluation of in vitro cytocompatibility, mechanical properties, and µCT visibility of GelMA-AuNP hydrogels. (**a**) Evaluation of the effect of AuNP size and concentration on cell metabolic activity (**: *p* < 0.01, ***: *p* < 0.001). Note that at 0.08 mM and 0.16 mM, for both 40 nm and 60 nm size, no differences were observed when compared to the control. (**b**) Assessment of the effect of AuNP size and concentration on mechanical properties of the GelMA hydrogel in a cytocompatible AuNPs concentration range; no differences between the experimental groups were observed. (**c**) The effect of AuNP size and concentration on radiopacity of GelMA-AuNP hydrogels. Blue marks the composition that showed the best X-ray attenuation value.

To enhance CT contrast of GelMA-based scaffolds, AuNPs, with different sizes and concentrations, were added to GelMA pre-polymer solution. Cellular metabolic activity of the L929 cell line significantly decreased for AuNP concentrations at 0.4 mM, for both 40 nm and 60 nm sized nanoparticles (Figure 2a). Therefore, mechanical testing and µCT scanning on GelMA-AuNPs was only subsequently performed in the cytocompatible concentration range (Figure 2b–c).

The results indicated that the addition of both 40 nm and 60 nm AuNPs did not significantly affect the compressive modulus of the GelMA hydrogels (Figure 2b). Afterwards, both GelMA and GelMA-AuNP hydrogels were scanned and compared regarding the changes in X-Ray attenuation (Figure 2c). The histogram of the gray values distribution showed a shift in the gray values for the hydrogel compositions labeled with the AuNPs. Particularly, a distinctive shift in the gray value and a sharper curve with an increased frequency were attained when 60 nm AuNPs were used with a concentration of 0.16 mM. Therefore, the GelMA-AuNPs formulation containing 0.16 mM 60 nm AuNPs was selected as showing the best performance in terms of mechanical properties, cytocompatibility, and enhanced radiopacity. Such GelMA-AuNPs hydrogel was used to prepare the GelMA-AuNPs scaffold for the osteogenic differentiation of MSCs.

### 3.2. 3D Printing of GelMA and GelMA-AuNP Scaffolds

**Figure 3:**
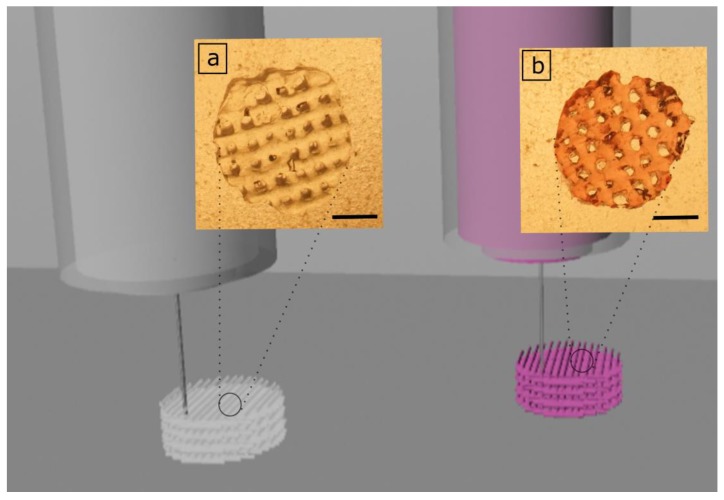
The 3D printed GelMA and GelMA-Au scaffolds. 3D printed GelMA (**a**) and GelMA-AuNP (**b**) scaffolds are indicated, and the addition of the AuNPs resulted in a slight pink color of the scaffolds (scale: 2 mm).

During the 3D printing process of hydrogels, it is crucial to attain stability of the 3D construct through permanent or temporary crosslinking methods. GelMA is a derivative of gelatin and shows similar thermoresponsive characteristics to gelatin hydrogels. Utilizing this characteristic, the GelMA and GelMA-AuNP solutions were cooled down to 20 °C and extruded onto a printing plate at 4 °C. The 3D constructs were printed with a printing speed of 18 mm/s, 2.3 bar extrusion pressure, and 200 µm layer thickness to ensure that 0°**/**90° oriented strands with a 1.2 mm distance were created (Figure 3).

### 3.3. In Vitro Evaluation of Osteogenic Differentiation of MSCs on GelMA and GelMA-AuNPs

**Figure 4:**
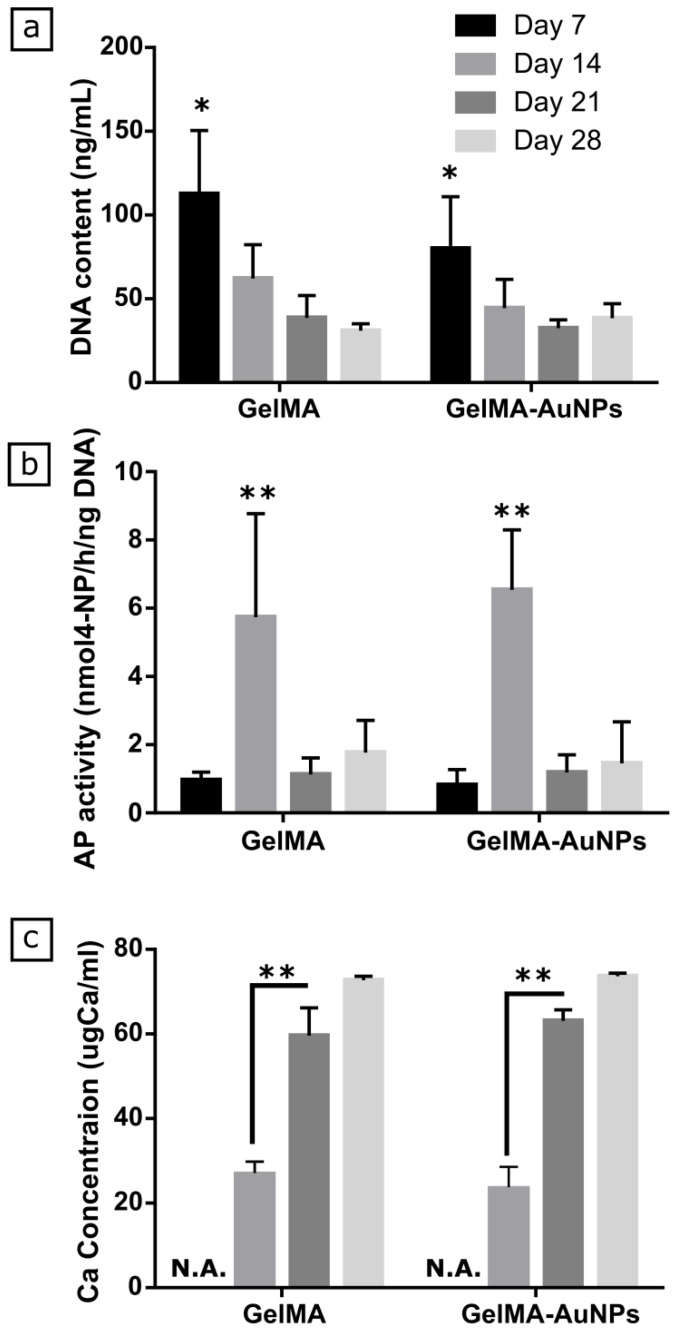
Colorimetric quantification of DNA content (**a**), alkaline phosphatase activity (**b**), and calcium deposition (**c**) through 28 days in vitro culture of MSCs over 28 days on GelMA and GelMA-AuNP scaffolds (*n* = 5, *: *p* < 0.05, **: *p* < 0.01).

DNA content, alkaline phosphatase activity, and Ca deposition on GelMA and GelMA-AuNP scaffolds were evaluated for 28 days (Figure 4). AP is an early osteogenic marker and in charge of mineralization of the extracellular matrix (ECM). Thus, we quantified the AP activity of MSCs to evaluate their osteogenic differentiation. As seen in Figure 4a, we did not observe any significant difference in the DNA content on GelMA scaffolds compared to GelMA-AuNP scaffolds over 28 days. On day 14, we attained a significant decrease in DNA content for both GelMA and GelMA-AuNP scaffolds and the decrease continued gradually over 28 days. We showed the AP activity of MSCs in Figure 2b, where no significant difference was observed between GelMA and GelMA-AuNP scaffolds, regardless of time points. We obtained significantly increased AP activity on day 14 for both GelMA and GelMA-AuNP scaffolds and after this, a decrease by day 21. For both hydrogel compositions, the calcium deposition on scaffolds began on day 14 and a significant increase in Ca accumulation was observed on the structures by day 21 (Figure 4c). The increased AP activity with the outset of Ca deposition on scaffolds on day 14 and a gradual increase in the Ca accumulation indicated that the MSCs were dedicated to osteogenic differentiation.

### 3.4. In Vitro Imaging of the 3D Printed GelMA and GelMA-AuNP Scaffolds in µCT

**Figure 5:**
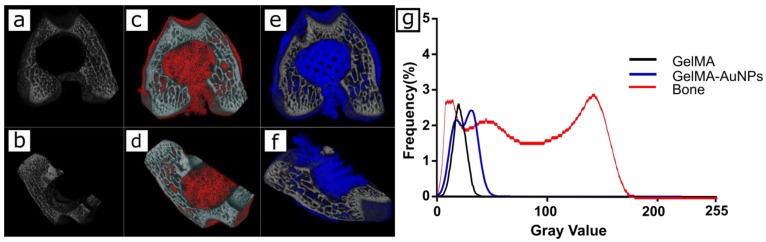
µCT tomograms of the empty rat condyle defect (**a**–**b**) and the inserted GelMA (**c**–**d**) and GelMA-AuNPs (**e**–**f**) along the transverse and coronal plane. (**g**) The radiopacity comparison of GelMA and GelMA-AuNP hydrogels to the bone tissue.

3D printed GelMA and GelMA-AuNPs were inserted into a condyle defect and scanned utilizing µCT. The acquired images were reconstructed; transversal (a,c,e) and coronal (b,d,f) cross-sections are qualitatively represented in Figure 5. It can be seen that visualization of 3D micro architecture was enhanced through encapsulation of the AuNPs. Furthermore, the histogram of the gray values distribution shows a distinctive shift in the gray value for 3D printed GelMA-AuNP scaffolds.

## 4. Discussion

GelMA-based hydrogels can serve as 3D model systems for different tissue engineering applications as their chemical, biological, and mechanical properties can be easily adjusted for the application of interest [29]. Our recent study showed the osteogenic differentiation of MSC, the homogeneous calcification through the GelMA hydrogels, and suitability for BTE applications [20]. After testing hydrogels containing different concentrations of GelMA, the composition with a 5% (w/v) ratio showed higher MSC attachment. Similarly, more homogeneous and significantly higher calcium deposition was also observed when 5% GelMA hydrogels were used. These results supported our choice of GelMA as a suitable material for BTE and also indicated the ideal concentration that needed to be used in further studies (i.e., 5% w/v). Even though the bulk GelMA hydrogels are suitable candidates to be used in bone tissue engineering, bone is a vascular tissue, whereby the integration of microchannels into GelMA hydrogels can further stimulate vascular network formation and increase the perfusion through the hydrogel [30]. Thanks to GelMA’s thermoresponsive characteristic, GelMA becomes a weak gel below its sol-gel transition temperature, which can be easily extruded through a nozzle for 3D printing purposes.

CT imaging gives highly substantial information regarding highly mineralized tissues (e.g., bone and teeth); however, the signals attained through high water-containing tissues or hydrogels are significantly low. Thus, even though hydrogels have been used for BTE applications, CT was only used to image the newly formed bone. [31,32,33] To overcome the challenge of imaging GelMA hydrogel via CT, it was hypothesized that the combination of GelMA with specific CA would improve the overall X-ray attenuation of the scaffold. Similarly, in previous studies, different iodinated hydrogel formulations were studied in the embolization of blood vessel applications, nucleus pulposus replacements, and as fiducial markers in gynecologic-cancer patients [34,35,36]. Hertig et. al. [37] suggested iodixanol combined fibrin hydrogels for endodontic applications. Their ex vivo preliminary experiments in human teeth showed that the radiopacity of the fibrin hydrogels was only sufficient for correct localization of the implant. Nonetheless, it was indicated that the characteristics of hydrogels dramatically changed due to the addition of the iodixanol CA. [37] In this study, it was crucial to preserve the physicochemical properties of the scaffolds and their cytocompatible characteristics. Thus, among different CAs, AuNPs have been utilized as a promising candidate due to their high X-ray attenuation, depending on their size and concentration [38]. The cytocompatibility of the gold nanoparticles has been questioned and evaluated in much previous research, as multiple studies have reported damaging effects of AuNPs regarding size and concentration. The cytotoxic effects of AuNPs can be observed as delays in doubling time, inhibition of proliferation, or increased apoptosis [38,39,40,41]. Therefore, the safety assessment and theranostic efficiency of AuNPs are crucially important for their applications in tissue engineering. It has been previously reported that the cytocompatibility of AuNPs increases with increased particle size. Nevertheless, Soenen et al. recently reported that toxic effects exerted by AuNPs are directly linked to their cellular uptake levels, so the influence of size may not be so straightforward, as well as the concentration [42]. Thus, firstly, we have evaluated the cytocompatibility in terms of the concentration and size of the AuNPs. The high concentration of AuNPs encapsulated in the GelMA scaffolds reduced the metabolic activity; consequently, the groups carrying 0.4 mM AuNPs were excluded from the study. In the range where AuNPs were not significantly affecting the metabolic activity of the L929s, the effectiveness of AuNPs was investigated as CA. AuNPs, besides their high X-Ray attenuation coefficient, have also been described to play a crucial role in vitro in the osteogenic differentiation of mesenchymal stem cells through the P38 mitogen-activated protein kinase pathway (MAPK) [14] and induce more bone formation (i.e., >12%) when compared to GelMA without AuNPs. [15] In line with the previous studies, here it was reported the AuNPs encapsulated in GelMA scaffolds did not hinder osteogenic differentiation of MSCs, and increased AP activity and calcification through the scaffolds were attained in GelMA-AuNP scaffolds without any significant difference compared to the GelMA scaffolds.

The AuNP sizes and concentrations used in this study were sufficient to increase the attenuation between GelMA hydrogel and the hard tissue mimicking constructs. Thanks to the apparent difference between bone and GelMA-AuNPs, the scaffold was clearly distinguishable through the construct; besides, the higher attenuation of GelMA-AuNPs compared to GelMA scaffolds can ensure better structural information regarding CT analysis and reconstruction. Most likely, higher AuNPs concentrations (> 0.4 mM) may give better CT attenuation; however, this may also dramatically affect the mechanical and biological properties of the GelMA.

## 5. Conclusions

With the recent progress in biomedical research, the monitoring of TE constructs possesses vital importance for translational research. Thus, the design of new TE scaffolds should not only address the need for biomaterials with optimal biological properties, but also focus on the imaging strategies required to follow its fate once implanted in vivo. In this study, a 3D printed GelMA-AuNPs scaffold with a high porosity and interconnectivity was designed. A cytocompatible AuNP size and concentration were evaluated to ensure enhancement in X-Ray attenuation without changing the physicochemical characteristics of the 3D printed GelMA scaffolds. These scaffolds showed good cytocompatibility for BTE application and evident osteogenic features. Moreover, 3D printed GelMA-AuNP scaffolds ensured better structural information regarding CT analysis and reconstruction. In conclusion, as the proof of concept, 3D printed GelMA-AuNPs scaffolds are good candidates for use in bone tissue engineering with enhanced visibility for µCT imaging.

## Supplementary Materials

The following are available online at http://www.mdpi.com/2073-4360/11/2/367/s1.
