# Supplementary material for: Enhancing X-ray Attenuation of 3D Printed Gelatin Methacrylate (GelMA) Hydrogels Utilizing Gold Nanoparticles for Bone Tissue Engineering Applications
Source: Polymers (Basel). 2019 Feb 20;11(2):367. doi: 10.3390/polym11020367 (PMC6419199; doi:10.3390/polym11020367)
Supplement: Supplementary file 1 [file polymers-11-00367-s001.pdf]

Supplementary Information

Physicochemical characterization of GelMA and GelMA-AuNP scaffolds

The physicochemical characterization of the GelMA and GelMA-AuNP scaffolds was investigated in terms of swelling from the wet state, the mass swelling ratio from the dry state, the gel fraction, and evaluation of the morphology with SEM. To evaluate the swelling from the wet state, hydrogel specimens (n = 3) were weighted (W_0) after UV crosslinking, put in a 12-multiwell tissue culture plastic (TCPS), and immersed in 2 mL 0.02% w/v sodium azide solution, used as a bacteriostatic agent. At established time points (t = 3, 24, 48 h), specimens were removed from water, gently swabbed with tissue paper, and weighted (W_t). The percentage weight variation (Swelling%) curves were obtained by plotting the average weight variation of samples at each time point as a function of time, calculated as shown in (1):

$$\text{Swelling\%} = (W_t - W_0)/ W_0 \times 100 \quad (1)$$

To evaluate the mass swelling ratio, anhydrous hydrogel specimens (n = 3) were weighted (W_d), put in a 12-multiwell tissue culture plastic (TCPS), and immersed in 2 mL 0.02% w/v sodium azide solution, used as a bacteriostatic agent. After 48 h, hydrogel samples were removed from the solution, gently swabbed with tissue paper, and weighted (W_s). The mass swelling ratio was calculated as shown in (2):

$$\text{Mass Swelling Ratio} = W_s/W_d \quad (2)$$

The hydrogel gel fraction represents the solid fraction retained by the hydrogel during the swelling. Anhydrous gelatin hydrogel specimens (n = 3) were weighted (W_d) and then swollen in 2 mL of 0.02% sodium azide solution for 48 h. After 48 h, hydrogel samples were removed from the solution and dehydrated under vacuum at 40 °C for two days. Then, the hydrogels were weighted (W_f) and the percentage gel fraction Gf was calculated as shown in (2):

$$\text{Gel Fraction \%} = W_f/ W_0 \times 100 \quad (3)$$

For SEM observation, the GelMA and GelMA-AuNP hydrogels were fixated in 2.5% glutaraldehyde in PBS for 2 h at room temperature and left for 12 h at 4°C. Samples were dehydrated with increased ethanol concentrations of 10, 25, 50, 70, 90, and 100%, for a total of 5 min for each concentration. The dehydrated samples were transferred to 50% HMDS diluted in ethanol for 20 min and transferred to a new 50% HMDS in ethanol solution for another 20 min. Samples were then transferred to 100% HMDS for 20 min, transferred to a new 100% HMDS solution, and then dried overnight. The dried samples were mounted on pins using double-sided carbon tape, and sputter-coated with 20 nm of gold. Specimens were examined using an SEM system (Phenom proX, PIK Instruments, Warsaw, Poland).

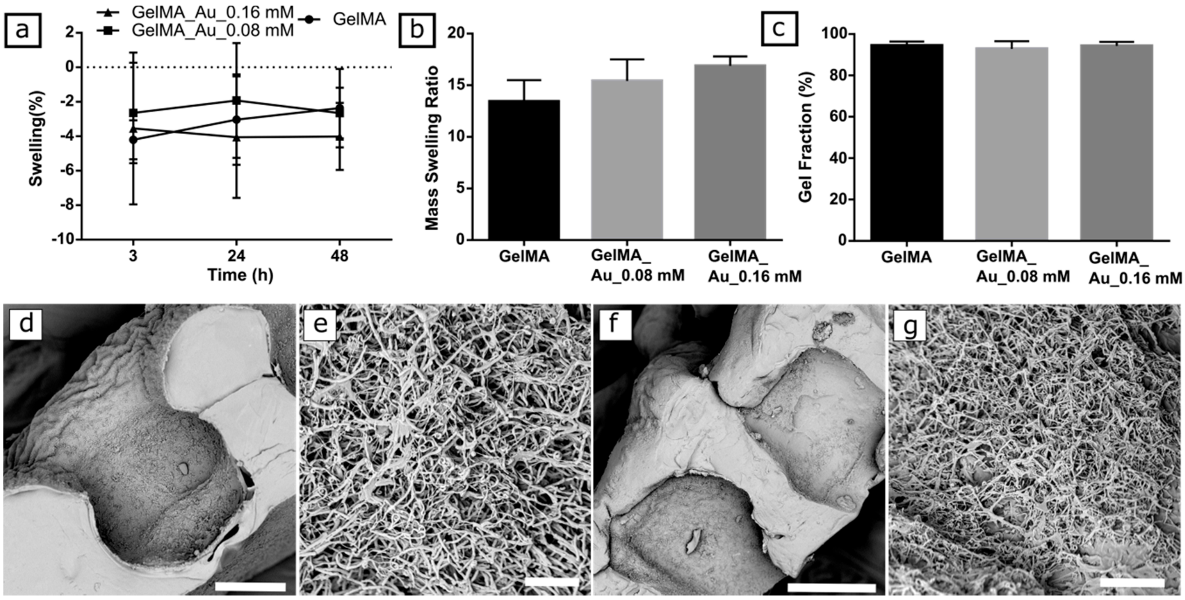

Figure S1. Physicochemical characterization of GelMA and GelMA-AuNP scaffolds. Swelling from the wet state (a), mass swelling ratio from dry state (b), gel fraction (c), SEM images of the 3D printed GelMA scaffold (d, scale bar: 200 um, magnification: 320 x; e, scale bar: 10 um, magnification: 5k x), and SEM images of the 3D printed GelMA-AuNPs scaffold (f, scale bar: 300 um, magnification: 270 x; g, scale bar : 20 um, magnification : 3k x).

Regarding our results, we obtained, with the given crosslinking parameters, scaffolds that were crosslinked and in a full swollen state. We performed gel fraction measurements to evaluate the solid fraction retained by the hydrogel during the swelling. We indicated that there was no significant weight loss in the 48 hours of incubation. Regarding the swelling of the hydrogels, the GelMA hydrogels showed similar swelling characteristics to those we have previously reported [1]. Even though the addition of the AuNPs slightly increased the swelling from the dry state of the scaffolds, the differences were not found to be statistically significant. Moreover, regarding the SEM observation, no significant differences were attained in the macro or micro structure of GelMA and GelMA-AuNP scaffolds.

1 Celikkin, N.; Mastrogiacomo, S.; Jaroszewicz, J.; Walboomers, X. F.; Swieszkowski, W. Gelatin methacrylate scaffold for bone tissue engineering: The influence of polymer concentration. J. Biomed. Mater. Res. Part A 2017.
